# An Uncommon Presentation of Autoimmune Hepatitis in a Child With Thalassemia Trait

**DOI:** 10.7759/cureus.38964

**Published:** 2023-05-13

**Authors:** Prashant B Khartade, Amar Taksande, Revat Meshram, Punam Uke

**Affiliations:** 1 Paediatrics, Jawaharlal Nehru Medical College, Sawangi Meghe, Wardha, IND

**Keywords:** immunosuppressive, hepatitis, thalassemic trait, jaundice, autoimmune

## Abstract

Autoimmune hepatitis (AIH) is quite rare in children. AIH is classified into two types based on the presence of autoantibodies: type 1 and type 2. The presentation of AIH varies, ranging from asymptomatic to acute or chronic hepatitis and occasionally fulminant liver failure. It can present at any age. In 20% of AIH cases, other autoimmune disorders might be present, such as diabetes mellitus and arthritis. A high index of suspicion is required for the early diagnosis of this condition. Pediatricians should consider the possibility of AIH in patients with jaundice once common causes are ruled out. The diagnosis is done on the basis of the presence of typical autoantibody titer, liver biopsy findings, and response to immunosuppressive medications. Some AIH patients may not respond to immunosuppressive therapy and may need a liver transplant. We present a case of a 12-year-old male child with thalassemia trait who was diagnosed with AIH.

## Introduction

Autoimmune hepatitis (AIH) is a chronic progressive inflammatory process caused by the presence of autoantibodies. AIH develops in genetically predisposed hosts and, once triggered, leads to an immune response targeting liver autoantigens. The triggering factors may include infections, drugs, molecular mimicry, and environmental toxins. Several human leukocyte antigens are associated with susceptibility to AIH. It is widely distributed globally and affects people of all ethnicities and ages, with a female preponderance. It can be present as acute hepatitis, which can mimic viral hepatitis or chronic liver disease, and may also manifest as fulminant liver failure. It progresses to liver cirrhosis unless properly treated. AIH is classified according to the presence of antibodies into two groups: type 1 and type 2 [1-2]. The patients may have nausea, anorexia, malaise, and pain in the abdomen. The clinical examination may not show any significant findings, but in chronic severe disease, the clinical features vary, including jaundice, hepatomegaly, splenomegaly, and stigmata of chronic liver disease. In addition to AIH, there may be other autoimmune disorders present in these patients, and hence pediatricians need to look for clinical features of other autoimmune disorders associated with AIH such as diabetes, thyroiditis, and celiac disease [3-5].

## Case presentation

A 12-year-old male child born of a non-consanguineous marriage, second by birth order, and a known case of thalassemia trait, presented with yellowish discoloration of eyes for one month, associated with fever initially. His jaundice had progressively increased to the extent that his skin had also become yellow, he was passing dark-colored urine, and his appetite had decreased. The family had sought medical advice at a local hospital, and they had investigated and symptomatically treated him there but no clear diagnosis had been reached; hence, he had been referred to a higher center. There was no distention or pain in the abdomen, no vomiting or loose motion, and no change in stool color. It was not associated with skin rash, bruises, or pruritus, and there was no history of joint or bone pain. There was no history of contact with a patient who had jaundice, no history of blood transfusion, and the patient was not on any medication. He had been delivered normally at full term, with no neonatal intensive care unit admission. He was developmentally normal for his age, his sibling was healthy, and there were no similar conditions in the family. There was no history of diabetes or arthritis or chronic diarrhea in the family. There were no deaths or abortions in the family history. Social history showed that they were living in a village with low socioeconomic status and there had been no recent travel. His vaccination was up to date.

On examination, he was underweight (21 kg less than the third centile), looked pale and deeply icteric, and was well hydrated. He was afebrile, with a pulse rate of 112/minute, respiratory rate of 24/minute, and blood pressure of 110/78 mmHg (normal heart rate: 60-100/minute, normal respiratory rate: 18-30/minute, systolic blood pressure: between 50th and 75th centile, and diastolic blood pressure: 90th centile); his SpO_2_ was 97% on room air. His abdomen was soft and non-tender, the liver was palpable, firm, and non-tender 4 cm below the costal margin with a span of 13 cm and the spleen was palpable 3 cm below the costal margin, with no ascites. The respiratory, cardiovascular, and central nervous system examinations were normal. His slit lamp examination revealed no Kayser-Fleischer ring. The musculoskeletal and skin examinations were also normal.

His laboratory workup was done, and his complete blood picture showed anemia. Peripheral blood smear showed microcytic anemia with anisocytosis, ovalocytes, elliptocytes, schistocytes, and poikilocytosis. His liver function tests were deranged [aspartate aminotransferase (AST), alanine aminotransferase (ALT), prothrombin time (PT), and international normalized ratio (INR) were elevated]. Erythrocyte sedimentation rate (ESR) and C-reactive protein (CRP) were raised. The platelet level was adequate. He was investigated for viral hepatitis, which was negative (hepatitis A, B, C, D, and E). Abdominal ultrasound was abnormal, revealing chronic parenchymal liver disease-hepatomegaly with diffuse coarse heterogeneous hepatic parenchymal eco texture and moderate splenomegaly. His ceruloplasmin level was within normal limits. Based on the above findings, the possibility of AIH was considered and he was evaluated for the same; his autoimmune workup was done, which was positive for ANA, anti-SMA, and anti-LKM antibodies. The patient was started on prednisolone 2 mg/kg/day in two divided doses and pantoprazole. An improvement was observed with the usage of ursodeoxycholic acid and vitamin K. His laboratory parameters were repeated after a week of therapy, which showed significant improvement.

## Discussion

Table 1 summarizes the AIH classification according to the presence of autoantibodies.

**Table 1:** AIH classification AIH: autoimmune hepatitis

Types of AIH	Autoantibodies present
Type 1	Antinuclear antibody
	Smooth-muscle antibody
	Antiactin antibody
	Autoantibodies against the soluble liver antigen and liver-pancreas antigen
	Atypical perinuclear antineutrophil cytoplasmic antibody
Type 2	Atypical perinuclear antineutrophil cytoplasmic antibody
	Antibody against liver cytosol type 1
	Antibody against liver-kidney microsomal type 3

AIH has a genetic basis; it is polygenic but does not have a specific pattern of inheritance. As described above, it has two types. Type 1 AIH is more common (60-70%), and it is characterized by the presence of ANA and anti-smooth muscle antibodies; it is more responsive to immunosuppressive therapy, and more common in adolescents than the adult population. AIH type 1 is sometimes associated with a mutation in the autoimmune regulator gene. This mutation may also present in autoimmune polyendocrinopathy syndrome, and hence clinicians need to search for other components of it such as candidiasis and ectodermal dysplasia [1-2]. Type 2 AIH is less common (about 20-30%), and it is characterized by the presence of anti-LKM antibodies; it is more severe and less responsive to immunosuppressive therapy. It has a bimodal distribution at a younger age of about 10 years and later at about the age of 40 years (can practically present at any age) and a variable clinical presentation [6-8].

Almost 30% of AIH patients are in the younger age group, which tends to have an acute presentation like infectious hepatitis. Hence, even in acute presentations, AIH should be suspected like in our case. It causes liver dysfunction with the elevation of AST, ALT, PT, and INR, while alkaline phosphatase (ALP) and gamma-glutamyl transferase (GGT) remain relatively normal. If they are elevated significantly, it is associated with sclerosing cholangitis. If sclerosing cholangitis is present, then clinicians have to look for the presence of overlap syndrome. The diagnosis of AIH is done after ruling out common causes of jaundice and based on the presence of a typical autoantibody profile, hypergammaglobulinemia, and typical histology on liver biopsy, and its response to immunosuppressive therapy confirms the diagnosis [3]. AIH type 2 causes a more hostile disease; it responds poorly to treatment and has a poor prognosis. A patient with anti-LKM antibody positivity tends to present with a high level of AST, ALT, and bilirubin and frequently goes into fulminant liver failure. Similar findings were present in our patient, with high AST, and ALT with high bilirubin [9].

The treatment involves immunosuppressive therapy comprising prednisolone with or without azathioprine. If the patient is intolerant to azathioprine or unresponsive to it, then other drugs can be added, such as mercaptopurine, cyclosporine, tacrolimus, mycophenolate mofetil, and monoclonal antibodies, e.g., rituximab. AIH is a progressive disorder that leads to cirrhosis of the liver; those who develop cirrhosis are at risk of developing hepatocellular carcinoma, and hence they need regular screening by imaging. The severity and progression of the disease are different in different ethnic groups. A study performed in Australia showed that Asian Australians who have AIH have more severe conditions and are unresponsive to treatment as compared to Caucasian populations in Australia. Similarly, in our case, the patient had raised enzymes, and his synthetic function was deranged at presentation [7,10-12].

AIH, like other autoimmune disorders, has a female preponderance globally, including in India; however, our patient was a male child. In India, AIH is considered very rare in children, and most pediatricians hardly see an AIH case in their lifetime of practice. AIH has a poor prognosis if it is not diagnosed and treated early. Almost 50% of the children affected by AIH have cirrhosis at presentation. Hence, early diagnosis and initiation of therapy are key to a better outcome. The diagnosis of AIH can be done by ruling out common causes such as viral hepatitis, and based on the presence of a typical antibody profile combined with classical findings on liver biopsy, and according to the response to immunosuppressive therapy [6].

In the present case, initially, we considered the common conditions causing jaundice, such as viral hepatitis, and inherited metabolic diseases such as Wilson's disease. When common causes of hepatitis ruled out the possibility of AIH considered, investigations were performed, which showed positive autoantibodies (ANA, ASM, and LKM). As described above, there are two different types of AIH, and our patient did not fit into any one type of AIH as he had ANA, ASM, and anti-LKM antibodies positive; hence, we diagnosed him as a case of AIH and started him on prednisolone 2 mg/kg/day; he responded to the treatment. Other drugs prescribed were pantoprazole and ursodeoxycholic acid, and vitamin K was also given. After starting on prednisolone, his clinical condition became better, his appetite improved, and his icterus gradually decreased. His repeat investigation showed remarkable improvement; his PT and INR became normal, AST and ALT declined by more than 50%, and his bilirubin level also decreased. So his response to prednisolone confirms our diagnosis as described above. Currently, this patient is on prednisolone, which will be gradually tapered off over the next six months and we will monitor his liver function and the side effects of steroids regularly. He was discharged home on prednisolone.

About 10-20% of AIH patients will need a liver transplant. The indication for transplant is a lack of response to medications or patients having complications of cirrhosis and presenting with fulminant liver failure. Even after a liver transplant, these patients will need regular monitoring for AIH because approximately 25% of transplant recipients can develop a recurrence of AIH. The treatment for a recurrence of AIH in transplant recipient patients is, strangely, prednisolone itself, and azathioprine together with cyclosporine in reduced doses has shown excellent results [2,5,8].

There are many reports associating the β-thalassemia trait with autoimmune diseases such as nephritis, diabetes, arthritis, fibromyalgia, and asthma. Thalassemia trait patients have an increased prevalence of rheumatic diseases. For example, one study showed a rheumatoid arthritis incidence of 6.4% in β-thalassemia trait subjects, compared with only 2.1% in the control population. There is one case report of a 10-year-old girl with a family history of systemic lupus erythematosus (SLE) who presented with acute-onset refractory seizures and was diagnosed with autoimmune anti-NMDA encephalitis [13-14].

There are two possible explanations for this increase in autoimmune diseases in thalassemia trait patients, one of which is that the hemoglobin β-chain locus is close to genes with profound roles in immune regulation, and the other relates to the reduced expression of hemorphins. Hemorphins have anti-inflammatory properties; they are closely linked to the suppression of inflammation and neutrophil migration. This reduced concentration can explain autoimmune susceptibility in those patients. Available sparse data indicate the reduced incidence of SLE in β-thalassemia heterozygotes; yet, if two conditions coexist, the SLE manifestations occur much more severely. This might be due to the close proximity of the beta-globin gene locus 11p 15.5 to the other eight important genes involved in the regulation of the immune system [13,15-16].

## Conclusions

Although AIH is rare among children, pediatricians must consider it in patients presenting with liver disease (acute or chronic) once the common causes are ruled out, in any gender despite the condition's female preponderance. AIH almost always progresses to cirrhosis, but early treatment can prolong survival and improve the quality of life.
